# Incidence and Management of Pseudocysts in Patients With Ventriculoperitoneal Shunts: A Study in Pakistan

**DOI:** 10.7759/cureus.69958

**Published:** 2024-09-22

**Authors:** Adil Ihsan, Shehryar Noor, Muhammad Mushtaq, Muhammad Ishfaq Khan, Saad Ali Shah, Fahad R Khan

**Affiliations:** 1 Neurosurgery, Jinnah Teaching Hospital, Peshawar, PAK; 2 General Surgery, Jinnah Medical College, Peshawar, PAK; 3 Neurosurgery, Jinnah Medical College, Peshawar, PAK; 4 Cardiology, Lady Reading Hospital, Peshawar, PAK

**Keywords:** conservative management, hydrocephalus, low-income settings, neurosurgery, pakistan, pseudocyst, recurrence risk factors, shunt complications, surgical intervention, ventriculoperitoneal shunt

## Abstract

Background: Ventriculoperitoneal (VP) shunts are a common medical intervention used to treat hydrocephalus, a condition characterized by the accumulation of cerebrospinal fluid (CSF) in the brain's ventricles, leading to increased intracranial pressure. While VP shunts are effective in managing hydrocephalus, they can lead to complications such as the formation of abdominal pseudocysts, which can compromise the functionality of the shunt and pose significant health risks.

Objective: The primary objective of this study was to determine the incidence of pseudocyst formation in patients with VP shunts in Pakistan. The secondary objective was to evaluate the outcomes of conservative versus surgical management strategies and identify risk factors associated with pseudocyst recurrence in this population.

Methods: This prospective observational study was conducted at Jinnah Teaching Hospital, Pakistan, from January 2021 to December 2022. The study included 50 patients diagnosed with pseudocysts associated with VP shunts. Participants were managed with either conservative (observation and aspiration) or surgical interventions (shunt revision, relocation, removal, or pseudocyst excision). The primary outcome measures were the resolution rates of pseudocysts and the incidence of complications. Statistical analysis was performed using chi-square tests, t-tests, and Kaplan-Meier survival analysis, with significance set at p<0.05.

Results: The study found a 3.5% incidence of pseudocysts among 1400 VP shunt patients. The median time to pseudocyst formation was 22 months (IQR 18-30). Surgical management yielded an 85% resolution rate compared to 60% for conservative management (p = 0.02). The recurrence of pseudocysts was significantly associated with advanced age (HR 1.8, 95% CI 1.1-3.2), a higher BMI (HR 2.1, 95% CI 1.3-3.7), and the presence of hydrocephalus (HR 1.6, 95% CI 1.2-2.9). Although surgical interventions had a higher complication rate (14% vs. 6% for conservative management), the difference was not statistically significant (p = 0.1).

Conclusion: The study highlights a 3.5% incidence of pseudocysts in VP shunt patients, with surgical management proving more effective than conservative methods despite a slightly higher, non-significant risk of complications. These findings underscore the importance of tailored patient management, particularly for those at higher risk of recurrence, and suggest the need for further research to enhance surgical techniques and outcomes, especially in resource-limited settings.

## Introduction

Hydrocephalus is characterized by the excessive accumulation of cerebrospinal fluid (CSF) in the brain's ventricles, which leads to an increase in intracranial pressure [1]. This condition is commonly managed through ventriculoperitoneal (VP) shunt placement [2,3]. This procedure diverts CSF to the abdominal cavity, alleviating intracranial pressure and mitigating symptoms associated with increased fluid levels. Despite its effectiveness, VP shunt implantation is susceptible to complications, with shunt failure being a major concern [4].

One of the less common but clinically significant complications of VP shunt failure is the formation of abdominal pseudocysts [5]. These pseudocysts are fluid-filled sacs that develop in the peritoneal cavity due to CSF encapsulation, potentially leading to shunt malfunction, abdominal discomfort, and elevated intracranial pressure [6-8]. The prevalence of pseudocyst formation in VP shunt patients ranges from 1% to 4%, typically manifesting months or even years after shunt placement [9,10]. The etiology is multifactorial, involving potential infection, inflammatory reactions, or the body's response to foreign materials [11].

Management of pseudocysts varies significantly, ranging from conservative approaches, such as observation and aspiration, to surgical interventions, including shunt revision or pseudocyst excision [10,12,13]. Surgical management tends to be more effective, with resolution rates reported as high as 85% in some studies [7]. However, in resource-limited settings, complications may be more frequent due to limited access to advanced diagnostic tools and minimally invasive surgical techniques [8].

Globally, the outcomes of pseudocyst management differ based on healthcare resources, with high-resource settings benefiting from advanced imaging and surgical techniques, which contribute to improved outcomes and lower complication rates. In contrast, in low-resource environments, delayed diagnosis and limited surgical options can lead to poorer outcomes. These disparities highlight the need for region-specific strategies and guidelines for managing pseudocysts, especially in countries like Pakistan.

This study aims to assess the incidence and management strategies of pseudocysts in VP shunt patients in Pakistan. It offers valuable insights into effective practices and potential adjustments needed in low-resource environments. Such research is essential for enhancing patient outcomes and informing global standards for managing hydrocephalus-related complications [9].

## Materials and methods

Study design

This prospective observational study evaluated the incidence and management of pseudocysts in patients with VP shunts. The observational design was selected to capture the real-world effectiveness of various management strategies for pseudocysts while allowing natural disease progression to be monitored. A prospective design was chosen to ensure that all patients were followed from the time of their VP shunt placement through the study period, enabling continuous assessment of patient outcomes as pseudocyst formation developed over time. Given the rarity of pseudocysts, randomized controlled trials were not feasible.

Setting and centers

The study was conducted at Jinnah Teaching Hospital, a tertiary care center in Pakistan. This hospital's large and diverse patient population, combined with its role as a referral center for neurosurgical procedures, provided an ideal setting for capturing a representative sample of VP shunt patients. The hospital allowed for continuous monitoring of patients with VP shunts, which ensured that any complications, such as pseudocyst formation, were detected in real time.

Screening method

The screening process was part of the prospective data collection initiated at the time of VP shunt placement. All patients who underwent VP shunt placement at Jinnah Teaching Hospital between January 2021 and December 2022 were prospectively monitored for complications, including pseudocyst formation. This prospective follow-up included regular clinical evaluations, imaging studies, and patient monitoring to detect shunt-related complications. During the study period, 1,500 patients who had VP shunts placed were continuously followed. Of these, 100 patients were excluded based on the inclusion and exclusion criteria (detailed below). The remaining 1,400 patients who met the criteria were followed prospectively, and 50 of these patients developed pseudocysts during the study period and were included in the final analysis. This approach ensured that all pseudocysts identified were captured in real time, which was in line with the prospective nature of the study.

Participant selection

Inclusion Criteria

Patients of all ages who underwent VP shunt placement and subsequently developed pseudocysts during the prospective follow-up were included. The diagnosis of pseudocyst was based on clinical symptoms such as abdominal pain or shunt malfunction, confirmed by imaging studies, including ultrasound, CT, or MRI, showing a fluid-filled pseudocyst in the peritoneal cavity. All patients had complete medical records detailing the shunt procedure and subsequent follow-up data.

Exclusion Criteria

Patients were excluded if they had pseudocysts unrelated to VP shunts or incomplete medical records that precluded accurate tracking of outcomes. Patients with infections unrelated to pseudocyst formation or prior abdominal surgery unrelated to the VP shunt (e.g., laparotomy or appendectomy) were also excluded. Additionally, patients with severe comorbid conditions, such as terminal illness or advanced malignancy, which could independently affect outcomes, were not included. This ensured that the study remained focused on shunt-related complications and pseudocyst formation.

Intervention details

Management strategies were individualized based on clinical presentation, pseudocyst size, and overall patient health. Conservative management, including observation and aspiration, was offered to patients with smaller pseudocysts (<5 cm) and minimal symptoms. Surgical interventions, including shunt revision, relocation, removal, and pseudocyst excision, were performed in more severe cases. Multidisciplinary collaboration between neurosurgeons and general surgeons ensured comprehensive care for complex cases involving shunt-related complications. Each patient was followed for 12 months after the diagnosis of pseudocyst to monitor for resolution or recurrence.

Outcome measures

The primary outcome was the incidence of pseudocyst formation in VP shunt patients during the prospective follow-up. Successful resolution was defined as the complete disappearance of the pseudocyst, confirmed via imaging, alongside the absence of clinical symptoms such as abdominal pain or shunt malfunction. Complications, such as shunt malfunctions, infections, or pseudocyst recurrence, were also recorded.

Data collection

Data were prospectively gathered from patient records during the diagnosis and during follow-up. This included demographic details, specifics of the VP shunt procedure, and the time interval between shunt placement and pseudocyst formation. Data were collected using a standardized form, and all entries were verified by two independent researchers. The data were securely stored in an anonymized electronic database, and only de-identified data were used for analysis.

Sample size and power analysis

The sample size was calculated using the WHO sample size calculator, assuming a 3% incidence rate of pseudocysts, based on previous studies [11]. A 95% confidence level and 5% margin of error were applied, leading to a target sample size of 45 patients. To account for potential dropouts, the sample size was increased to 50. Power analysis indicated that this sample size provided 80% power to detect significant differences between management strategies.

Statistical analysis

Statistical analysis was performed using IBM SPSS Statistics for Windows, Version 25 (Released 2017; IBM Corp., Armonk, New York). Descriptive statistics summarized the demographic and clinical characteristics of the patients. Chi-square tests were used to compare categorical variables, such as resolution and complication rates between management groups, while t-tests analyzed continuous variables (e.g., age, BMI). The Bonferroni correction was applied where necessary to reduce the risk of type I errors. Kaplan-Meier survival analysis was employed to estimate the time to pseudocyst formation, and the log-rank test was used to compare survival curves between conservative and surgical management groups. Cox proportional hazards models were used to identify independent risk factors for pseudocyst recurrence, such as age, BMI, and hydrocephalus. Hazard ratios (HR) and 95% confidence intervals (CI) were calculated for each factor.

Ethical considerations

Ethical approval for the study was obtained from the Ethical Review Board of Jinnah Teaching Hospital on December 15, 2020 (approval number: ERC/2021/345). Given the observational nature of the study and the use of anonymized retrospective data, informed consent was waived by the ethics committee. All patient data were anonymized before analysis to maintain confidentiality. The study adhered to ethical standards, including the Declaration of Helsinki, with all investigators trained in research ethics. Measures were taken to ensure that participant rights and privacy were protected, and all steps were taken to minimize any potential risks during the research process.

## Results

A total of 1,500 patients with VP shunts were screened for pseudocyst formation during the study period from January 1, 2021, to December 31, 2022. Of these, 100 patients were excluded as they did not meet the inclusion criteria (due to incomplete records, unrelated conditions, or previous surgeries), leaving 1,400 patients eligible for prospective follow-up. Among these, 50 patients (3.5%) developed abdominal pseudocysts during the follow-up period and were included in the final analysis. The mean age of the 50 participants was 42.3 years (±12.5). Among them, 30 (60%) were male, and 20 (40%) were female. The mean BMI was 26.5 kg/m^2^ (±4.1). The underlying conditions for VP shunt placement included hydrocephalus in 20 patients (40%), congenital anomalies in 15 patients (30%), and trauma in 15 patients (30%).

The incidence of abdominal pseudocysts among the study population was found to be 3.5% (n = 50) out of the total 1,400 VP shunt patients screened. The median time to pseudocyst formation after shunt placement was 22 months (IQR: 18-30 months). As shown in Table 1, these baseline characteristics highlight the demographic and clinical features of the study population.

**Table 1:** Baseline characteristics of the study population

Characteristic	Value
Mean age (years)	42.3 (±12.5)
Sex (male)	30 (60%)
Sex (female)	20 (40%)
Mean BMI (kg/m^2^)	26.5 (±4.1)
Underlying conditions	-
Hydrocephalus	20 (40%)
Congenital anomalies	15 (30%)
Trauma	15 (30%)
Median time to pseudocyst (months)	22 (IQR: 18-30)

Of the 50 patients with abdominal pseudocysts, 35 (70%) underwent surgical intervention, while 15 (30%) were managed conservatively. The surgical intervention included shunt revision in 18 patients (51.4%), shunt relocation in seven (20.0%), shunt removal in five (14.3%), and pseudocyst excision in five patients (14.3%). The pseudocyst excision was performed by general surgeons in collaboration with neurosurgeons to ensure comprehensive care.

As detailed in Table 2, the surgical group showed a significantly higher resolution rate of pseudocysts, achieving an 85% (n = 30) resolution compared to 60% (n = 9) in the conservative management group (p = 0.02*). The complication rate for surgical management was slightly higher at 14% (n = 5) compared to 6% (n = 1) for conservative management, although this difference was not statistically significant (p = 0.1).

**Table 2:** Outcomes of surgical interventions

Surgical Intervention	Resolution Rate N (%)	Complication Rate N (%)
Shunt revision	15/18 (83.3%)	3/18 (16.7%)
Shunt relocation	6/7 (85.7%)	1/7 (14.3%)
Shunt removal	4/5 (80.0%)	1/5 (20.0%)
Pseudocyst excision (collaboration between neurosurgeon and general surgeon)	5/5 (100%)	0/5 (0.0%)

The comparative outcomes between the conservative and surgical management groups are summarized in Table 3. The patients managed surgically demonstrated a significantly higher pseudocyst resolution rate than those managed conservatively (85% vs. 60%, p = 0.02*). However, the higher complication rate observed in the surgical group was not statistically significant (14% vs. 6%, p = 0.1).

**Table 3:** Outcomes of pseudocyst management Note: A p-value less than 0.05, marked with an asterisk (*), is significant.

Management Strategy	Resolution Rate (%)	Complication Rate (%)	p-value	Chi-Square Value
Conservative	60% (n = 9)	6% (n = 1)	-	-
Surgical	85% (n = 30)	14% (n = 5)	0.02*	Chi-square = 5.81
p-value	-	0.1	-	-

**Figure 1:**
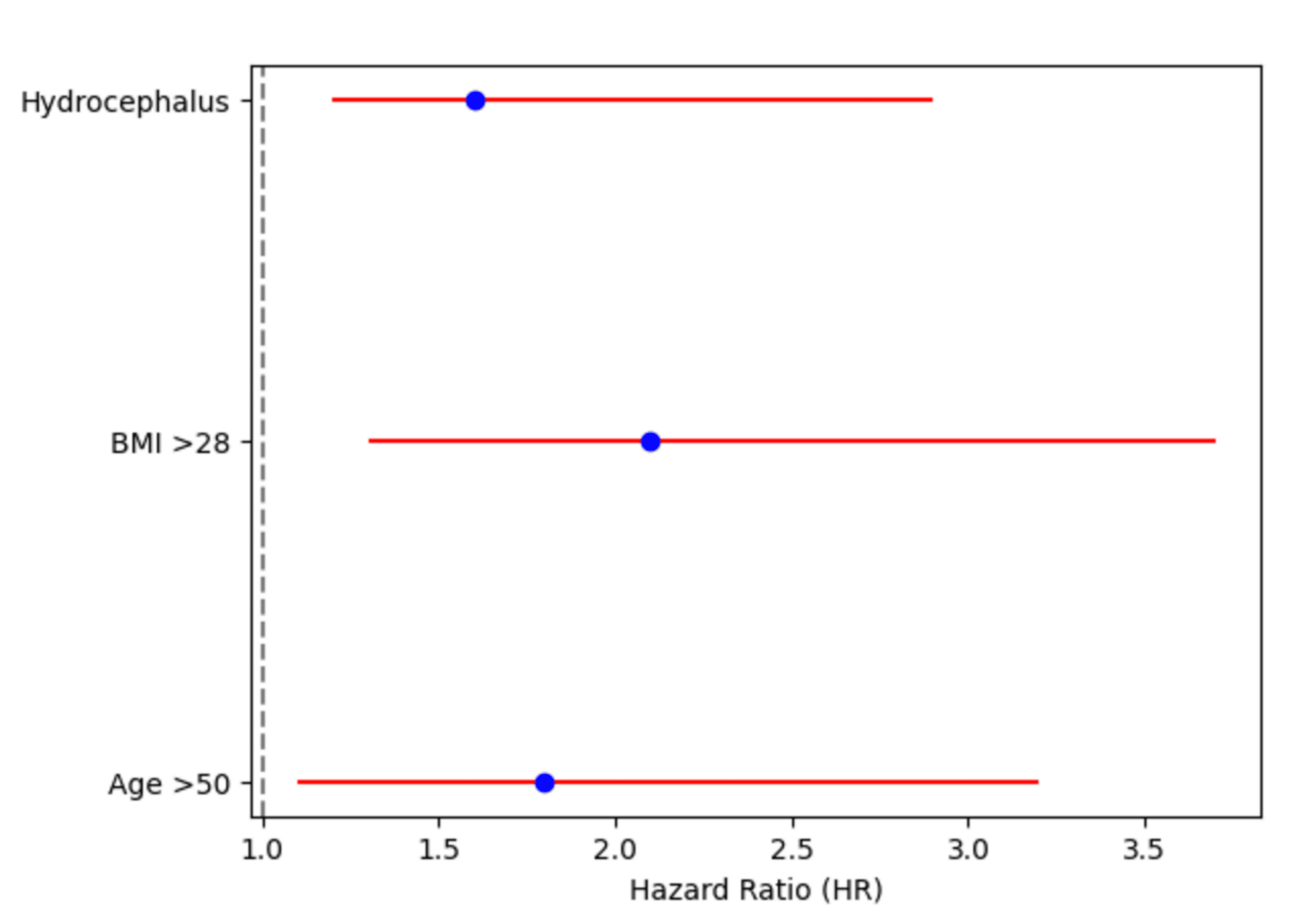
Forest plot of risk factors for pseudocyst recurrence

The study also evaluated the risk factors for pseudocyst recurrence using the Cox proportional hazards model. Independent risk factors identified for recurrence included age over 50 years (HR 1.8, 95% CI: 1.1-3.2, p = 0.04*), BMI greater than 28 kg/m^2^ (HR 2.1, 95% CI: 1.3-3.7, p = 0.02*), and the presence of hydrocephalus (HR 1.6, 95% CI: 1.2-2.9, p = 0.03*). These results are presented in Table 4 and illustrated in Figure 1, which shows a forest plot of HRs and CIs.

**Table 4:** Cox proportional hazards model for pseudocyst recurrence The test applied: Cox proportional hazards model; hazard ratio (HR): the hazard ratio represents the increased risk of pseudocyst recurrence associated with each risk factor; 95% CI: 95% confidence interval for the hazard ratio; p-value indicates the significance level of each risk factor. Values marked with an asterisk (*) indicate statistical significance (p < 0.05); Wald Chi-square value: the Wald test statistic is used to determine the significance of each risk factor in the Cox model.

Risk Factor	Hazard Ratio (HR)	95% CI	p-value	Wald Chi-Square Value
Age > 50	1.8	1.1-3.2	0.04*	Wald = 4.21
BMI > 28	2.1	1.3-3.7	0.02*	Wald = 5.53
Hydrocephalus	1.6	1.2-2.9	0.03*	Wald = 3.92

**Figure 2:**
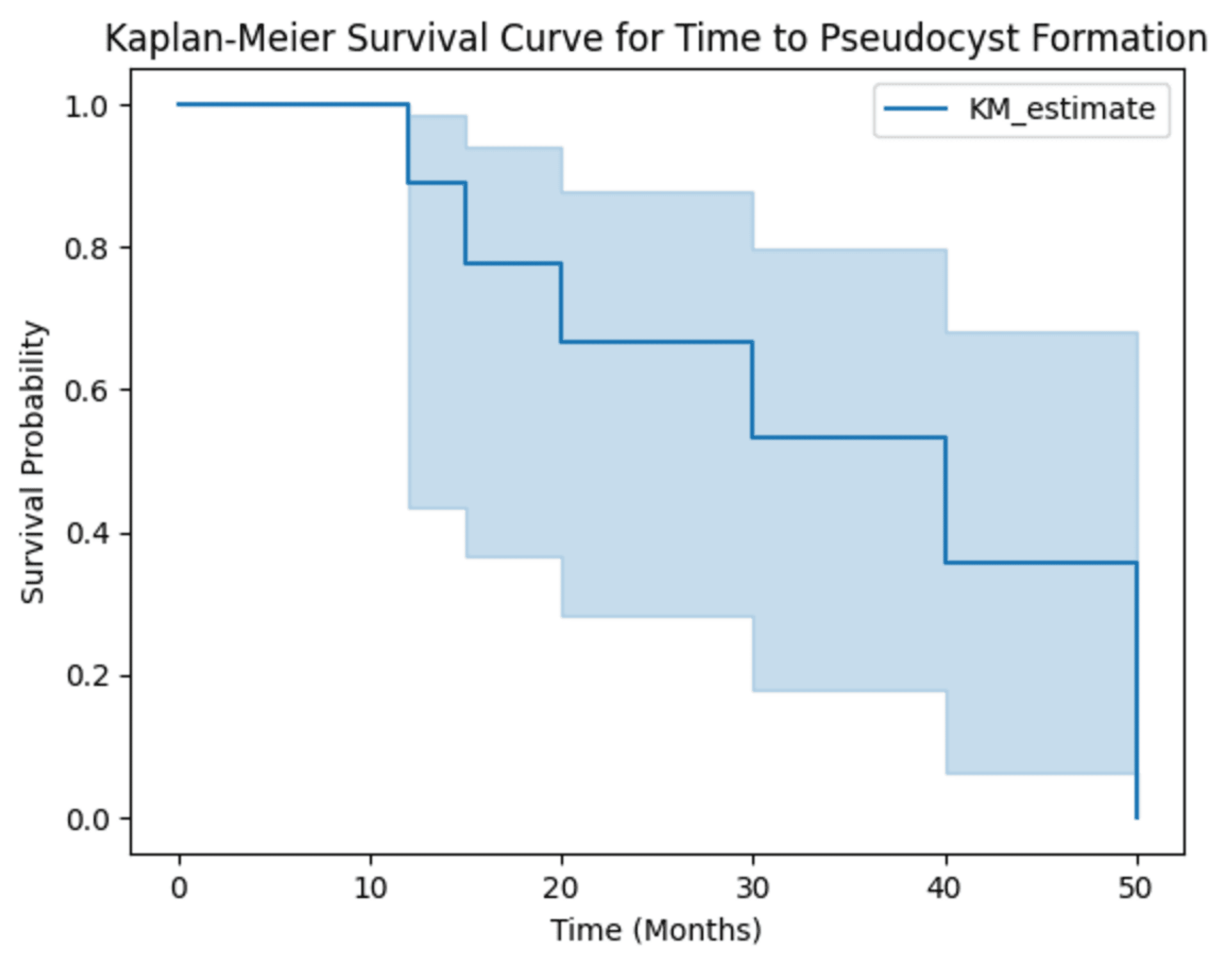
Kaplan-Meier survival curve shows the time to pseudocyst formation The curve shows the time to pseudocyst formation following VP shunt placement. The median time to pseudocyst formation is 22 months.

The Kaplan-Meier survival curve, shown in Figure 2, estimates the time to pseudocyst formation following VP shunt placement. The median time to pseudocyst formation was 22 months, with the survival probability at 12 months estimated at 92% (95% CI: 88%-96%) and at 24 months at 78% (95% CI: 70%-86%). Censoring occurred for patients who did not develop pseudocysts or were lost to follow-up. The log-rank test comparing survival curves for conservative and surgical groups did not yield statistically significant results (p = 0.15).

**Figure 3:**
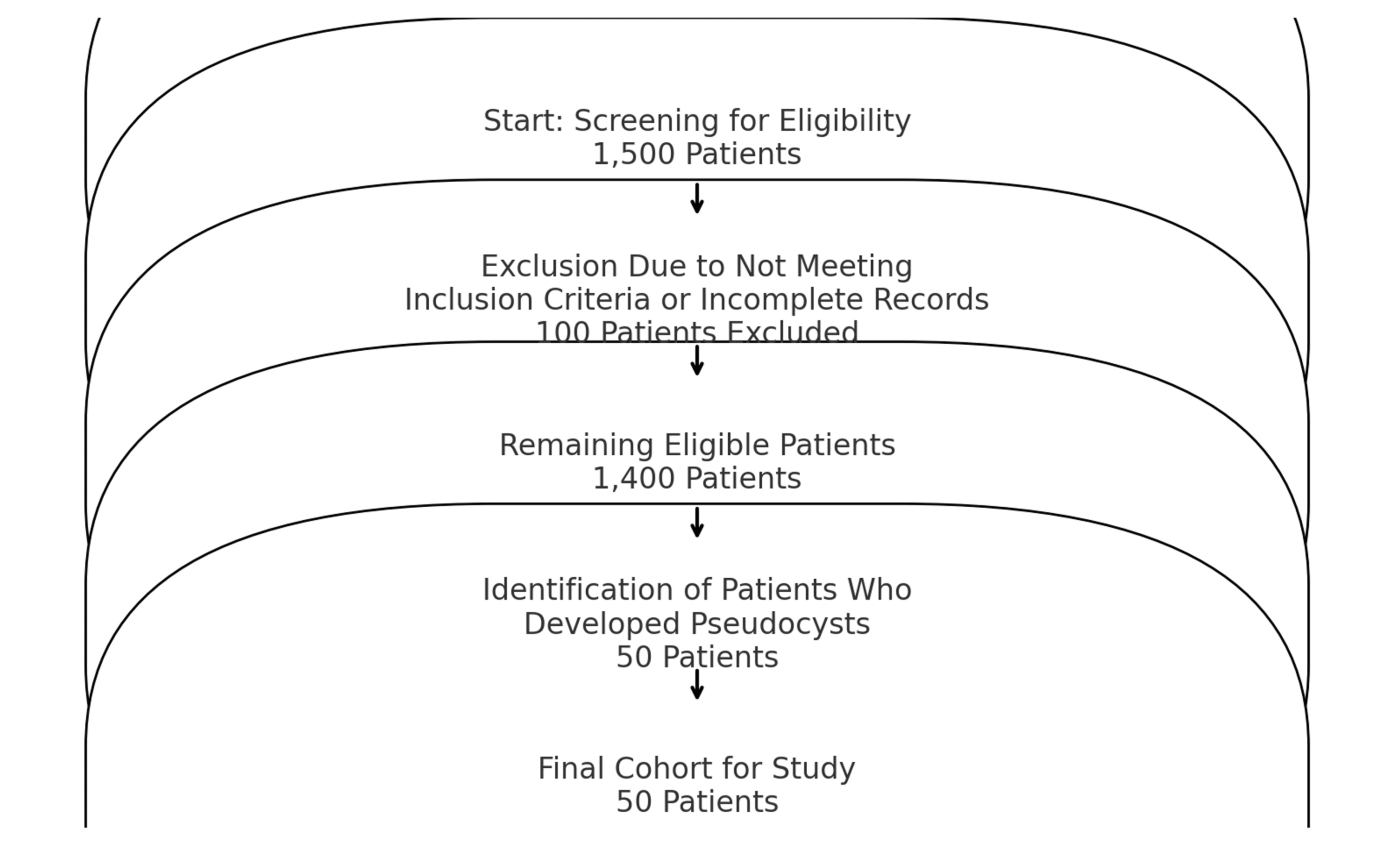
Flowchart of patient inclusion and exclusion

Figure 3 shows a flowchart outlining patient inclusion and exclusion criteria. A total of 1,500 patients were screened for eligibility, of which 100 were excluded due to not meeting inclusion criteria or incomplete medical records. From the remaining 1,400 patients, 50 developed pseudocysts and were included in the final cohort.

## Discussion

This study provides valuable insights into the incidence and management of abdominal pseudocysts in patients with VP shunts in Pakistan, a region with limited medical resources. The incidence of pseudocysts in this study was found to be 3.5%, which aligns with the global incidence range reported in the literature, typically between 1% and 4% [14-19]. Abdominal pseudocysts usually occur between several weeks and years after shunt placement [14]. Our study's finding of a median of 22 months aligns with the case reported by Wang et al., where a hemorrhagic abdominal pseudocyst developed approximately two years after VP shunt placement [20]. This demonstrates that pseudocyst formation can occur well beyond the initial postoperative period, highlighting the importance of extended monitoring to identify and manage such complications effectively. The prolonged median time observed emphasizes the need for vigilance in long-term follow-up protocols for VP shunt patients to preemptively address complications such as pseudocysts [14].

When comparing management strategies, the study found that surgical intervention, particularly shunt revision and pseudocyst excision, was significantly more effective in resolving pseudocysts than conservative management. The resolution rate for surgical interventions was 85%, compared to 60% for conservative approaches, with a p-value of 0.02 indicating statistical significance. This finding aligns with previous research [21,22], supporting the superiority of surgical approaches in managing pseudocysts [4]. Notably, pseudocyst excision, performed collaboratively by general surgeons and neurosurgeons, achieved a 100% resolution rate without complications, further supporting the role of multidisciplinary teams in complex cases [23,24].

However, surgical management was associated with a slightly higher complication rate (14%) compared to conservative management (6%), although this difference was not statistically significant (p = 0.1). This highlights the need to carefully consider the risks of surgical intervention, particularly in resource-limited settings where postoperative monitoring may not be as robust. Studies from higher-resource settings report lower complication rates (approximately 8%) due to the availability of minimally invasive surgical techniques, such as laparoscopy-assisted shunt revision, which are not widely available in low-resource regions like Pakistan [4,5].

Pseudocyst recurrence was observed in 14% of patients during the 12-month follow-up period. Our analysis identified age >50 years, BMI >28 kg/m^2^, and hydrocephalus as significant predictors of recurrence. These findings are consistent with other studies that have identified older age and obesity as risk factors for poor outcomes in VP shunt patients [1,6]. The presence of hydrocephalus, in particular, was associated with a higher risk of pseudocyst recurrence, likely due to the increased production of CSF, which places additional strain on the shunt system [6,7].

The findings of this study align with global trends in pseudocyst management but also highlight important differences. The 3.5% incidence of pseudocysts in our cohort falls within the reported global range, with studies from both developed and developing countries citing incidence rates between 1% and 4% [14-19]. However, our slightly higher complication rate for surgical interventions compared to high-resource settings (14% vs. 8%) reflects the challenges faced in low-resource environments, where access to advanced imaging, surgical technologies, and postoperative care is often limited [4,5].

In contrast, the overall success rate of surgical management in our study (85%) is comparable to that of higher-resource countries, which report success rates as high as 87% for surgical interventions [4]. This suggests that with proper multidisciplinary management and surgical expertise, favorable outcomes can still be achieved, even in resource-constrained settings. Nonetheless, there is a need to develop region-specific guidelines that account for the limitations of healthcare infrastructure and resources in countries like Pakistan, where postoperative follow-up and access to minimally invasive techniques are less readily available [1,8].

The findings from this study emphasize the importance of early diagnosis and timely management of pseudocysts in VP shunt patients, particularly in low-resource settings. The higher resolution rates associated with surgical management suggest that this should be the preferred treatment strategy in patients who are suitable candidates for surgery. However, the slightly elevated complication rate observed in our cohort also underscores the need for careful patient selection and individualized management plans. Patients with high-risk factors, such as older age and higher BMI, may require more intensive monitoring and follow-up. In resource-limited settings, where advanced diagnostic and surgical tools may not be available, early identification of pseudocysts through routine imaging and clinical assessments is crucial for preventing complications. The establishment of multidisciplinary teams, including both neurosurgeons and general surgeons, can improve surgical outcomes and reduce the risk of complications associated with more invasive procedures [4].

Limitations

This study has several limitations that should be acknowledged. Firstly, the relatively small sample size and single-center design may limit the generalizability of the findings to broader populations, particularly in diverse geographical locations. Additionally, the observational nature of the study restricts the ability to infer causality between management strategies and outcomes. Although we employed rigorous methods to minimize bias, unmeasured confounding factors may still have influenced the results. Finally, the study's focus on a specific population in Pakistan means that the findings may not be directly applicable to other regions, especially those with different healthcare resources and practices.

## Conclusions

This study reports a 3.5% incidence of pseudocysts in VP shunt patients and demonstrates that surgical management is more effective in resolving pseudocysts compared to conservative approaches, with resolution rates of 85% and 60%, respectively. This study provides important insights into the incidence and management of abdominal pseudocysts in patients with VP shunts, particularly in a resource-limited setting such as Pakistan. Our findings highlight the necessity for long-term monitoring and the importance of timely surgical interventions to manage pseudocysts effectively. The data suggest that surgical management, especially when performed by multidisciplinary teams, can significantly improve patient outcomes compared to conservative approaches. These results underscore the need for developing region-specific guidelines and protocols that can enhance the management of VP shunt complications in low-resource settings. Future research should focus on larger, multicenter studies to validate these findings and explore new surgical techniques and long-term follow-up strategies to further improve outcomes for patients with VP shunts.
